# Measures of public attitudes towards science: A scoping review

**DOI:** 10.1177/09636625261458106

**Published:** 2026-07-12

**Authors:** Jessica E. Hughes, Kimberley Norris, James D. Sauer, Aaron Drummond, Holly Emery, Emilia Hawkey, Victoria J. Heinrich, Paul Schokman, Matthew A. Palmer

**Affiliations:** 1 School of Psychological Sciences, University of Tasmania, Launceston, Australia

**Keywords:** attitudes towards science, scoping review, PRISMA, scale development

## Abstract

The role that attitudes towards science plays in science acceptance has received attention in recent years, yet little is known about the ways that public attitudes towards science are conceptualised and measured. To address this, our review sought to identify instruments developed to measure attitudes towards general science or the scientific method for use with adult populations that had been subjected to psychometric analyses. The review protocol was pre-registered and methodology adhered to the Joanna Briggs Institute guidelines and PRISMA extension for scoping reviews. Title and abstract screening of 16,658 articles by two independent reviewers identified 783 articles for full-text screening, resulting in 31 articles (29 separate instruments) for inclusion. Psychometric properties were summarised; variations in conceptualisations of attitudes towards science and adherence to best-practice recommendations for scale development were identified. Overall, this research contributes to our ongoing understanding of attitudes towards science and the measurement of this important construct.

## 1. Introduction

Society has been, and will continue to be, faced with important matters of global consequence. Climate change is a challenge that requires an urgent and collective response in order to avoid catastrophic repercussions (IPCC, 2023). The recent COVID-19 global pandemic that resulted in an estimated 17.2 million deaths globally provides another example (Sachs et al., 2022). Science can provide evidence-based guidance to address these problems; however, some recent evidence suggests that public acceptance of science is eroding (Gauchat, 2012; Hamilton and Safford, 2021 cf. Miller et al., 2024, for evidence that at least some of the decline may be explained by reliance on polling instruments). Irrespective of the rate of change, understanding this decline in acceptance of science and devising strategies to arrest the decline and/or improve science acceptance is of critical importance. In light of this, there has been growing attention around the impact of positive attitudes towards science on public acceptance of science. Promisingly, the literature demonstrates that positive attitudes towards science predict greater acceptance of, and behaviour aligning with, the views of the scientific community – for example, acceptance of the scientific consensus on climate change, support for scientific funding, and support for policy (Drummond et al., 2016; Drummond and Fischhoff, 2017; Hughes et al., 2023; Motta, 2018). This is encouraging: attitudes are often malleable, so promoting more positive attitudes towards science might be one approach to boosting public acceptance of science, and improving societal outcomes (Hughes et al., 2023).

Given that there are many ways of thinking about attitudes towards science – and that some attitudes might be more closely linked than others to public acceptance of science – one important step is to understand different ways that attitudes towards science can be conceptualised and measured. With this in mind, the aim of the present study was to review different scales that have been developed to measure attitudes towards science among the public.

### Conceptualising and measuring attitudes towards science

Attitudes towards science started receiving increased attention from around the 1950s when social surveys identified a decreasing number of students interested in undertaking science subjects at school (Aiken and Aiken, 1969). However, it was not until the 1970s that interest in the public understanding of science started growing (Miller, 1983). Much of the research regarding public attitudes towards science has focused on attitudes relating to specific topics in science and technology (e.g. climate change, vaccinations, biotechnology), rather than the broader construct of science more generally (e.g. the use of science to know and understand the world, the benefits of science to society, or the trustworthiness of scientific results).

Attitudes towards science have been conceptualised in various ways in the literature. With a focus on attitudes towards science held by non-experts (i.e. people without specific scientific expertise), a useful distinction can be drawn between educational and non-educational settings. In the context of education research, attitudes towards science often index students’ feelings about science in general, learning science specifically, engaging in science activities, and careers in science (Toma and Lederman, 2020; Tytler and Osborne, 2011). Research on such topics has featured strongly in the education literature, resulting in a number of robust instruments to assess attitudes towards science specifically in an educational context. Indeed, there have been several reviews conducted of instruments used in education settings (e.g. Blalock et al., 2008; Toma and Lederman, 2020).

For public attitudes towards science outside of educational settings, conceptualisations tend to focus upon the intersection between science and society (i.e. beyond the school environment) and the ways in which citizens interact with science in daily life. Generally, attitudes towards science can be described as the values, beliefs, and feelings an individual holds about science as an institution, the processes of science, scientists, and the role of science in society (Gardner, 1975; Osborne et al., 2003). Research exploring the public’s attitudes towards science has continued to develop over recent years but, compared to work in educational settings, relatively little attention has been paid to the ways in which these attitudes have been measured.

### Scale development and quality

Although best-practice recommendations for scale development exist (e.g. see American Educational Research Association (AERA) et al., 2014; Boateng et al., 2018; DeVellis, 2012), many scales are not constructed in line with these practices (Clark and Watson, 2019; Pardo and Calvo, 2002). Broadly, these recommendations include ensuring the construct is defined or clarified, generating items through deductive (using pre-existing literature) or inductive (using interviews or focus groups) methods, item reduction, and performing tests of dimensionality, reliability, and validity (see e.g. Boateng et al., 2018; DeVellis, 2012 for a more detailed description of these steps). Although scale development is an iterative process (Clark and Watson, 2019), attention to methodological recommendations has been varied, and the literature documents a lack of systematic processes across several domains within the psychological sciences (Barry et al., 2014; Flake et al., 2017; Flake and Fried, 2020). In this area, some scales lack a sound theoretical basis, some have not established evidence of dimensionality or validity, and some use a direct measure of the construct (e.g. a single item asking participants to what extent they trust science, which may save time but not take into consideration the complexities of constructs; Besley and Tiffany, 2023; Pardo and Calvo, 2002; Reif et al., 2024). Indeed, questionable measurement development practices have arguably contributed to the current replication crisis in psychological science (Flake and Fried, 2020; Simmons et al., 2011). In turn, this obscures our understanding of important topics, and may, in itself, erode public trust in science, and result in the devaluation of science. Hence, pursuing research using measurement tools with sound psychometric properties is desirable to ensure research practices are rigorous, outcomes are valid, and that meaningful replication can occur (Flake and Fried, 2020). In order to do this, we need to understand how public attitudes towards science are being measured, so that gaps in current practices and knowledge may be addressed.

### The present study

The aim of this scoping review was to identify instruments that have been used in empirical research to measure public attitudes towards science among non-expert, adult populations, and to summarise the psychometric properties of these instruments. To the best of our knowledge, no comprehensive reviews have been undertaken for this purpose. This review will assist future research by minimising duplication in the development of new instruments and enhance our understanding of constructs underlying attitudes towards science. In turn, this will allow for future work to be conducted to improve the psychometric properties of existing scales, and enhance the rigour and transparency of research.

Although reviews of scales measuring attitudes towards science have been undertaken in school settings (e.g. see Blalock et al., 2008; Toma and Lederman, 2020), as noted throughout, our focus was to identify and summarise instruments used in public settings (i.e. non-expert, adult populations), including their psychometric properties. We aimed to systematically map the available evidence relating to this topic, identify key characteristics of instruments, understand how research is being conducted in this field, and highlight gaps in current practice. Given the broad, exploratory nature of our research question, our aims best aligned with the guidelines for scoping reviews. This is in contrast to systematic reviews, which require a narrow question, and which focus on providing a comprehensive appraisal of the quality of current evidence and providing conclusions based on this evidence (e.g. regarding the efficacy of an intervention; Munn et al., 2022; Peters et al., 2015).^1^ The systematic methodology employed in scoping reviews allows for a comprehensive survey of the literature and minimises bias, compared to traditional narrative reviews. Scoping reviews can also be useful for understanding whether a more narrow systematic review is warranted (Munn et al., 2022; Peters et al., 2015, 2022; Tricco et al., 2018).

## 2. Method

This scoping review adhered to the Joanna Briggs Institute (JBI) guidelines (Peters et al., 2015) and was reported in accordance with the PRISMA Extension for Scoping Reviews (PRISMA-ScR; Tricco et al., 2018).

### Protocol and registration

A protocol was registered via PROSPERO on 3 June, 2022 (Registration number *CRD42022315463*).

### Eligibility criteria

Eligibility criteria are detailed in Table 1, with additional information regarding definitions for criteria listed in Table 2. As criteria 4 and 6 indicate, to be included, a study had to address a population consisting of non-expert adults aged 18 years or older. The concept was to identify instruments that had been developed, evaluated, or refined and where psychometric properties had been reported, and in the context of attitudes towards general science or the scientific method. Studies were required to be reported in the English language, and published in peer-reviewed journal articles.

**Table 1.** Full-text screening exclusion hierarchy.

Step in hierarchy	Exclusion criteria
1.	Non-English language.
2.	Non-peer reviewed journal (e.g. books, conferences, abstracts, short communications, dissertations).
3.	Non-original research article (e.g. review).
4.	Included non-adult participants (i.e. aged less than 18 years).
5.	Wrong study design (i.e. study objective is not to develop/evaluate/refine the instrument or is post hoc design)
6.	Only included expert sample (see definition of ‘public’).
7.	Only in an educational context (see definition of ‘education setting’).
8.	Did not include psychometric analyses (see definition of ‘psychometric analyses’) of the instrument/s’ properties.
9.	Instrument (see definition of ‘instrument’) does not meet definition of attitudes (see definition of ‘attitudes’).
10.	Instrument (see definition of ‘instrument’) does not meet definition of science (see definition of ‘science’).

**Table 2.** Definition of scoping review exclusion criteria terminology.

Term	Definition
Public	Non-experts (i.e. not specific professionals with specialised qualifications targeted for their expertise).
Education setting	Not related to attitudes towards the personal teaching or personal learning of science (studies with university populations are acceptable).
Instrument	Quantitative or mixed methods (quantitative and qualitative, but not only qualitative) scale or questionnaire.
Attitudes	Positive or negative evaluative judgements (feelings, thoughts, beliefs, intentions) towards an attitude object (i.e. ‘science’ as per the definition specified). Attitudes do not include only knowledge/literacy.
Science	Construct relates to science in general (e.g. science, or scientific inquiry/methods in general). This does not include specific areas/topics of scientific application, specific institutions involved in science, or scientists.
Psychometric analyses	One or more of the phases of scale development and validation (see Boateng et al., 2018) have been reported. For scale evaluations/refinement, need to have reported at a minimum tests of item reduction/dimensionality.
Adults	All participants in the study are aged 18 years or older.

Following the first round of title and abstract screening and prior to commencing full-text screening, further refinement of definitions and eligibility criteria were made, as is common and acceptable in scoping reviews (Peters et al., 2015). The definition of ‘public’ was refined to exclude expert populations, for example, those with specialised skills and qualifications needed to perform a role and targeted in the research for their expertise (e.g. doctors, engineers). We refined this criterion to better align with our intention to identify instruments used within the general population, and we did not anticipate search results relating to specific professions. Second, to avoid confusion during full-text screening, we refined ‘science’ to mean relating to science in general or to the scientific method, which excluded scales that predominantly assessed specific topics of science (e.g. chemistry), institutions (e.g. universities), or scientists. Two subsequent refinements were made to criteria during full-text screening to clarify and align with the original intentions of the review. First, as we intended to focus our review on dedicated scale development, to satisfy the ‘study design’ inclusion criterion, studies that did not develop a scale but only evaluated (e.g. examined whether the scale was an adequate measure of a construct) or refined (e.g. developed a short-form) a scale needed to have performed at a minimum tests of item reduction or dimensionality. For example, if a study used a scale but only reported Cronbach’s alpha, this would not be included. Further, articles that exclusively used a post hoc, exploratory methodology were excluded (e.g. where large survey data was explored; but see, for example, Miller (2004) for some analyses of large survey datasets). Second, to satisfy the ‘science’ criterion, the overall construct needed to meet this criterion; that is, if some items of the instrument met the definition, but others did not, and the overall construct the instrument was intended to measure did not meet the definition of science, it would be excluded. For example, some scales included a small number of items relating to science in general, but the majority of items were relating to scientists.

Given the broad nature of this research area, eligibility was not limited to particular journals. As this was the first review of this type to our knowledge, we also chose not to limit the search by publication year to ensure comprehensiveness. We included only original articles published in the English language in peer-reviewed journals.

### Information sources and search strategy

The search strategy was informed, in part, by previous systematic reviews that identified instruments measuring attitudes towards science^2^ in education settings (see Blalock et al., 2008; Toma and Lederman, 2020). The present search strategy necessarily deviated from these previous search strategies, particularly in the terms relating to *attitudes towards science*. This occurred because prior knowledge and pilot searches indicated that, for public attitudes towards science, there is diversity in the way in which these terms are described. For example, *attitudes towards science* may be described as *trust in science* or *science interest*, which would not be identified by the term attitud*. A research librarian was consulted in the formulation of the search strategy, which was reviewed by four members of the research team.

Pilot searches were undertaken in March 2022. The first formal search was undertaken on 4 April 2022, with a subsequent updating search on 6 September 2024. Searches were undertaken in Web of Science, Scopus, and PsycINFO. Abstract and title searches included a combination of search terms across (1) science, (2) attitudes, and (3) scale development terms, and search terms to exclude articles relating only to an education context or non-adult populations (e.g. ‘*primary school*’, ‘*middle school*’, child*). Asterisks (*; e.g. scien*) were used to capture variations of the terms (e.g. *scientific, science, scientists*). Search queries were constructed based on the requirements of each database (see Supplemental Table 1 for a complete list of search terms). Following recommendations, backwards searching of reference lists, handsearching, and consultation with experts also occurred to identify any relevant additional sources not identified by the formal search (Arksey and O’Malley, 2005).

### Selection of sources of evidence

Identified records (*n* = 36,371) were imported into the web-based software platform, Covidence (www.covidence.org). Duplicate records (*n* = 19,343) were removed automatically, with any additional duplicates removed manually throughout screening (*n* *=* 350). Title and abstract screening (*n* = 16,658) was undertaken by the primary reviewer (the first author) and a second independent reviewer. Articles deemed relevant were then moved to full-text screening (*n* = 783) and their eligibility assessed by the primary reviewer and a second independent reviewer. Reviewers independently pilot screened 20 titles and abstracts, and five full-texts, which were then discussed in meetings for the purpose of resolving any uncertainties in the inclusion criteria. No masking of authors or journal names occurred. Disagreements on whether to include or exclude a record were resolved between the two reviewers; in cases where the two reviewers were unable to resolve disagreements, a third independent reviewer was engaged. Full-text screening was guided by an exclusion hierarchy (Table 1). There were 31 articles included. The overall Cohen’s kappa, which is a measure of internal consistency (Cohen, 1960), for title and abstract screening was .51, and for full-text screening was .47, which is considered moderate (Landis and Koch, 1977). Overall agreement for title and abstract screening was 96%, and for full-text screening was 94%.

### Data charting process and data items

Data extraction followed a deductive approach (Pollock et al., 2023), whereby information about instruments’ properties was categorised according to recommendations for scale development (AERA et al., 2014; Boateng et al., 2018; DeVellis, 2012), and thematic interpretation of measured constructs was informed by the literature (e.g. Gardner, 1975; Klopfer, 1971, 1976; Osborne et al., 2003). The first author independently extracted and organised the data using a customised data extraction form, which detailed: name of instrument, number of items, response scaling, response options, article title, author(s), journal, date of publication, population (number, age), study setting (sample type, country), and the reported presence of psychometric properties (theoretical background, item development, item reduction, dimensionality, reliability, content validity, criterion validity, and construct validity). A random sample (*n* = 12) was assessed by a second reviewer, and there were no disagreements. Where detail of the instrument’s items was insufficient to make a determination of eligibility, authors were contacted by email; articles were excluded if a response was not received (*n* = 4). This occurred because the screening process had revealed that, occasionally, authors’ description of a scale appeared to meet inclusion criteria (e.g. authors specified the scale was a measure of attitudes towards science), but once items were reviewed, it became apparent that it did not (e.g. items related to experts or specific topics of science).

Consistent with the aims of this review, information reported by authors relating to the psychometric properties of instruments was recorded, but not analysed or formally evaluated. Information was categorised based on recommendations for scale development (e.g. Boateng et al., 2018; DeVellis, 2012). Theoretical background was determined to be present if authors specified a theoretical basis or clear definition of the construct the instrument intended to measure (e.g. specifying relevant theory, stating a clear definition, detailing the relationship of the construct with other constructs; DeVellis, 2012). Item development was determined to be present if a process of item development was reported (e.g. items written by authors, adapted from previous scales). Item reduction was determined to be present if authors reported a process of testing/reducing the number of items to optimise measurement. Reliability was determined to be present if any form of reliability was reported (e.g. internal consistency, test–retest). Dimensionality was determined to be present if any form of dimensionality testing was reported (e.g. exploratory factor analysis (EFA), confirmatory factor analysis (CFA), principal components analysis (PCA), item response theory (IRT)). Content validity was determined to be present if authors reported any evaluation of items by experts or target population. Criterion validity was determined to be present if authors reported relationships with direct measures of the target phenomenon or gold standard measures. Construct validity was determined to be present if authors reported any tests of construct validity (e.g. convergent validity, discriminant validity, differentiation by known groups, or tests of association with existing measures; Boateng et al., 2018; DeVellis, 2012).

### Critical appraisal of individual sources of information

We did not undertake a formal evaluation of quality or risk of bias, given this is more applicable to systematic reviews where evidence is evaluated and recommendations for practice made, which is not the objective of scoping reviews (Grant and Booth, 2009; Tricco et al., 2018). We chose to take a similar approach to Toma and Lederman (2020), whereby the specific analyses of the instruments’ psychometric properties undertaken were documented, as opposed to making a judgement about the quality of the instruments. This approach allows future researchers the opportunity to make their own evaluations of the quality of instruments and to undertake additional analyses as deemed necessary, given scale development is an iterative process (Clark and Watson, 2019).

## 3. Results

### Selection of sources of evidence

**Figure 1.**
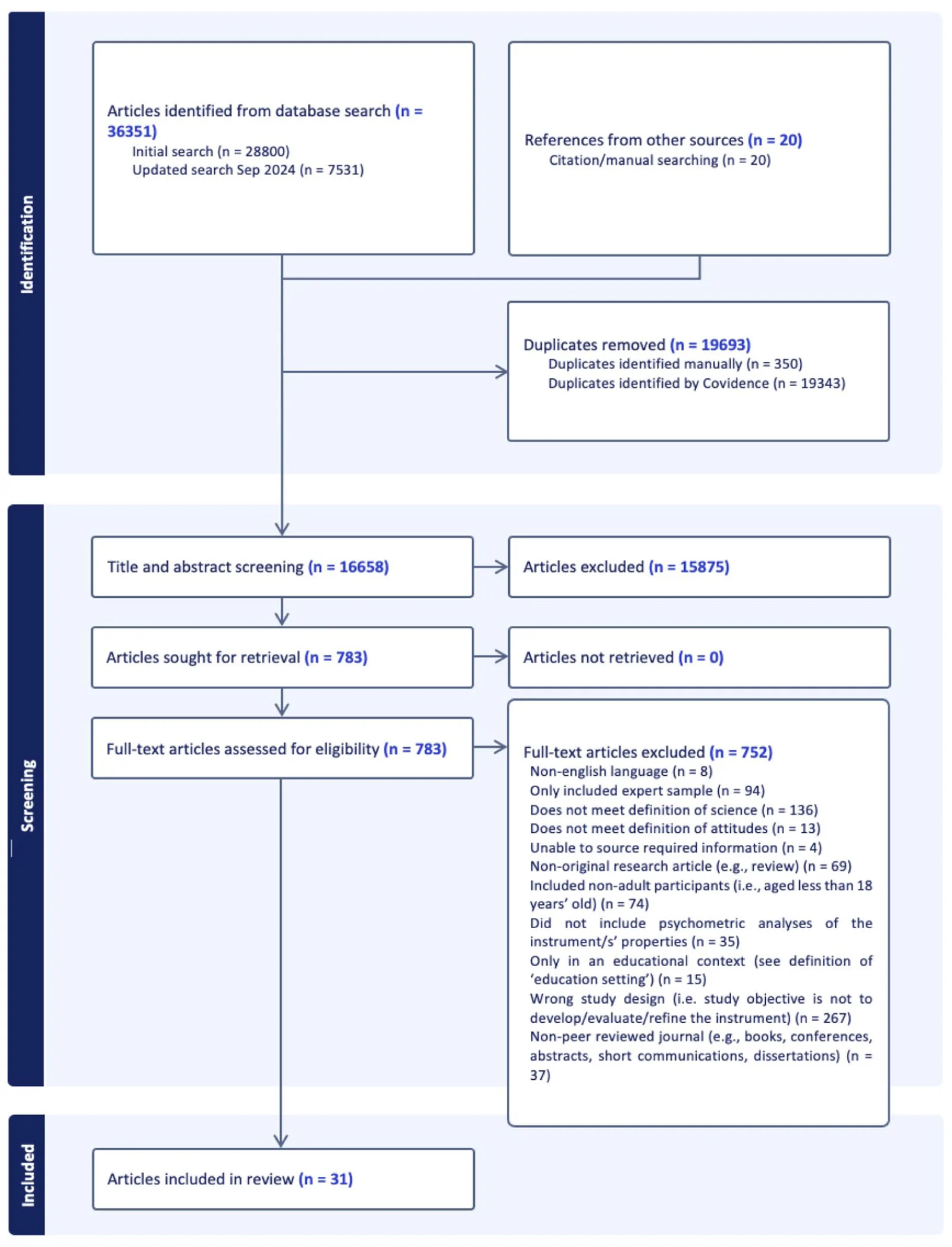
PRISMA flow chart summarising the number of articles included at each stage of the scoping review (identification, screening, inclusion).

Figure 1 details the number of articles included at each stage and reasons for exclusion.

### Characteristics of sources of evidence

Appendix 1 Table 3 contains an overview of the characteristics of instruments identified in this review. From the 31 articles reviewed, 29 separate scales were identified. Some articles included more than one instrument meeting criteria, and some articles evaluated the same instrument, so there were 34 cases in total. The majority of studies were conducted on populations in North America (United States, *n* = 13; Canada, *n* = 1), with studies also occurring in Europe (France, *n* = 1; Germany, *n* = 2; Italy, *n* = 1; Poland, *n* = 2; Serbia, *n* = 1; United Kingdom, *n* = 2), South America (Argentina, *n* = 1), Oceania (Australia, *n* = 1; New Zealand, *n* = 1), and Asia (Japan, *n* = 1). Africa was not represented. Participant country was not reported in six instances (either within studies reported in the article or the entire article). Population sample reported included general public (*n* = 27), University students (*n* = 5), and citizen science volunteers (n = 1). One article reported no details about population.

### Characteristics of instruments

The number of items reported ranged from 2 to 60 items. Twenty-two instruments used Likert-type scales, 1 used a Thurstone scale, and 6 either used a variety of response scaling or response scaling was not specified/reported. Many instruments reported using a don’t know/neutral response option, but the wording varied (e.g. ‘difficult to say’, ‘draw’, ‘neutral’, ‘neither agree or disagree’, ‘unsure’, ‘don’t know/unsure’).

### Constructs

There was diversity in the constructs measured by the identified instruments. It should be noted that the items on many scales indexed multiple themes, so these results reflect themes relating to individual items. Six broad themes were identified and interpreted with consideration of theory (e.g. Gardner, 1975; Klopfer, 1971, 1976; Osborne et al., 2003): *Scientific inquiry as a way of thought, science and its inter-relationships with society, nature of science, interest in science, scientific conduct*, and *trust in science*.

*Scientific inquiry as a way of thought* relates to the use of science and scientific research to know and understand the world, make decisions, and solve problems. For example, the Belief in Science Scale includes items such as ‘Science is the most efficient means of attaining truth’ and ‘The scientific method is the only reliable path to knowledge’ (Dagnall et al., 2019). Klopfer (1971, 1976) coined the term *scientific inquiry as a way of thought* in his taxonomy relating to attitudes towards science in an educational context. In Klopfer’s taxonomy, (*scientific) inquiry as a way of thought* could be defined by a value for objectivity, rationality, and critical thinking in the context of science.

*Science and its inter-relationships with society* relates to the role of science in society, perceived risks and benefits of science, and the influence of science. Examples of relevant questions are ‘Scientific findings and technological developments enrich the human society’ from the Science-Appreciating Scale (Kawamoto et al., 2013), and ‘The development of science brings about problems to humanity’ from the Valuation of Science and Technology scale (Vaccarezza, 2007). Klopfer (1971, 1976) conceptualised *science and its inter-relationships with society* as a sub-category of the broader science construct, arguing that there is a need to differentiate between science as a source of knowledge and in terms of the way science influences society, culture, and policy. Klopfer also argued for distinct sub-categories relating science as an organised enterprise and the characteristics of scientists as people.

*Nature of science* relates to views about what science is and how various aspects of scientific processes work. Instruments measuring nature of science reported here reflected beliefs, feelings, and attitudes about various aspects of science and in our view did not constitute simply a measure of knowledge. Though topics relating to *nature of science* might typically be thought to reflect understanding of science, the instruments that we reviewed and included often assessed this using Likert-type response scales which reflected participants’ degree of agreement with statements. Assessing in this way, as opposed to a dichotomous true/false response, allows for individuals to express stronger or weaker agreement with ideas. For example, the Nature of Science instrument (Lombrozo et al., 2008) asks, ‘Scientific theories are not just guesses’. While, theoretically, this statement is true, an individual with a particularly sceptical attitude towards science may disagree, which may be reflective of a scepticism towards science, rather than knowledge of scientific processes. Other items relating to this theme include ‘Science could prove the existence of supernatural beings like God’ and ‘Scientific research is not influenced by society and culture because scientists are trained to conduct “pure”, unbiased studies’ (Lombrozo et al., 2008).

*Interest in science* relates to interest in participating in science events, consuming science-related media, or undertaking scientific endeavours. For example, the Science Curiosity Scale includes items assessing how closely participants attend to news relating to scientific research or discoveries, the frequency of attendance at a science or technology museum, and the frequency of reading books relating to scientific research or discoveries (Kahan et al., 2017). In comparison to the other themes identified, measuring *interest in science* has the advantage of incorporating behavioural questions, whereby participants can not only self-report the degree to which they think science is interesting, but also whether they engage in science-based activities, which strengthens validity. This is indeed observed in the Science Curiosity Scale. While incorporating behavioural measures may be possible when measuring other themes, it would appear to be somewhat less straightforward.

*Scientific conduct* relates to perceived justice, norms, motives, and morality of science and scientific research. For example, the Norms of Science instrument assesses views about how scientific research should be conducted, scientific findings should be used, and scientists should conduct themselves across 14 items, such as ‘Scientific findings should be available to everybody everywhere in the world’ (Lewandowsky and Oberauer, 2021). This theme extends beyond Klopfer’s (1971, 1976) sub-category of science related to *scientists as people*, which reflected more of an understanding that scientists are unique, relating oneself to scientists, and reflecting on one’s own conduct. However, the *scientific conduct* theme identified here also taps into a moral and ethical value that people hold about the way science should be conducted, used, and shared. In addition to a moral component, it could be argued that a certain level of knowledge would be required for respondents to engage with these topics. For example, it might be assumed that a degree of explicit teaching about ethics would be necessary for an individual to understand what ethical conduct should look like.

*Trust in science* relates to perceptions about whether science can be believed, such as the extent to which the products of science are certain, accurate, reliable, or valid. For example, the Credibility of Science Scale assesses perceived accuracy, usefulness, and motives in relation to scientific methods, information, and the institution of science across 6 items, such as ‘People don’t realize just how flawed a lot of scientific research really is’ (Hartman et al., 2017). Our criteria excluded instruments that were exclusively related to scientific institutions or scientists. However, many *trust in science* instruments contained some questions relating to institutions and scientists, as well as questions about science in general. Indeed, the themes of *scientific conduct* and *trust in science* provide a good example of the complexity of measuring attitudes towards science. On one hand, there is a perception of science that relates to the processes and outcomes of science. On the other hand, science also involves people and institutions (as outlined by Klopfer, 1976), which individuals may appraise discretely, for example, by applying moral and ethical considerations. Therefore, understanding the interrelatedness of these aspects may be more complex than originally thought. For example, it is plausible that an individual may be willing to accept a piece of scientific evidence as true, yet may hold more negative beliefs about particular institutions due to political influences (e.g. see Hamilton and Safford, 2021). Understanding how such issues may overlap is important for ensuring instruments truly tap the intended construct.

While some scales appeared to assess a unitary construct, other scales combined items that arguably target separate constructs. For example, the Scientific Attitude Instrument used by Price and Lee (2013) combines items relating to interest in science-based media and activities, self-reported knowledge of science, and personal application of science knowledge in daily life. A number of scales contrasted science with other topics, such as religion or technology. Relatedly, some instances of double-barrelled items were noted. For example, an item from the Science-Appreciating scale (Kawamoto et al., 2013) states ‘Science provides us not only practical usefulness, but also intellectual pleasure’, which assesses the separate constructs of usefulness and pleasure or interest in the same item.

### Psychometric properties

Appendix 2 Table 4 provides an overview of reported psychometric properties of the identified instruments. A theoretical background was reported in 28 cases. A description of the process of item development was reported in 29 cases. Item testing and reduction processes were reported in 14 cases. Tests of dimensionality were reported in 27 cases. The most commonly reported technique for testing dimensionality was CFA (*n* = 14), but other techniques were also reported, including EFA (*n* = 11), PCA (*n* *=* 7), IRT (*n* = 5), structural equation modelling (SEM; *n* = 1), and other techniques (*n* = 3). Many cases employed a combination of techniques. Tests of reliability were reported in 30 cases. By far the most common method used was Cronbach’s alpha for internal consistency reliability, which was reported in 23 cases. Other procedures were less commonly reported, including IRT (*n* = 5), McDonald’s omega (*n* = 4), Pearson’s *r* (*n* = 1), test–retest (*n* = 1), and other techniques (*n* = 1). Reliability estimates ranged from .60 to .95. Evidence of content validity was reported in 6 cases. Evidence of criterion validity was reported in 12 cases. Evidence of construct validity was reported in 29 cases. Construct validity was the most commonly reported test of validity. It should be noted that the quality of reported scale-development procedures varied, for example, whether sample size recommendations for EFA were followed or whether recommended cut-off ranges were applied. However, as noted above, quality was not assessed in this review.

## 4. Discussion

### Summary of evidence

Twenty-nine instruments were identified in this review. The psychometric properties reported by authors are summarised below. Though a formal quality assessment was outside the scope of this review, informally it was identified that a number of instruments may not satisfy best-practice recommendations for scale development (AERA et al., 2014; Boateng et al., 2018; DeVellis, 2012). For example, two instruments contained only two items, which may not reflect sufficient validity for complex constructs (Allen et al., 2022; Eisinga et al., 2013; Emons et al., 2007; Smith et al., 2000).

### Constructs

The constructs measured in the scales identified by this review reflected the complexity of measuring attitudes towards science. Six broad themes were identified: *scientific inquiry as a way of thought, science and its inter-relationships with society, nature of science, interest in science, scientific conduct*, and *trust in science.* Items on some instruments indexed multiple themes; however, the literature would suggest these are conceptually discrete (e.g. see Klopfer, 1971, 1976; Osborne et al., 2003). For example, though likely related, an individual does not have to enjoy science or be interested in consuming scientific media to believe that it is the best way to solve complex problems (Drummond et al., 2016; Hughes et al., 2023). Indeed, attitudinal components of science are distinct from science knowledge (Allum et al., 2008; Wynne, 2006). Further, contrasting science with other ideological factors, such as religion, assumes a dichotomy; however, these ideological conflicts may exist in some domains (e.g. where there are moral implications, such as stem cell research), but not others (e.g. where scientific opinion does not conflict with religious teachings, such as the nature of electrons and atoms; Evans, 2011; McPhetres and Nguyen, 2018). Thus, it is important that (a) it not be assumed that attitudes towards science reflects a unitary construct, and (b) the subconstructs of focus in measurement have a sound theoretical basis (Osborne et al., 2003).

### Item and scale development

The majority of cases reported evidence of theoretical background (82%; e.g. a clear definition of the construct of interest), and item development procedures (85%; e.g. deductive or inductive methods). This is positive, because having a clear understanding of what is being measured guides item development. However, it should be noted that the review of individual items revealed that these did not always appear to align with the construct as defined. Fewer cases reported processes of item reduction (41%). Only 18% of cases reported content validity, for example, evaluation by experts or target population. This is of concern, as ensuring an instrument measures what it intends to is fundamental to scale development. Further, assessing content validity is important so that items are interpreted by participants as intended, and to ensure that items reflect an accurate measure of the construct by unbiased assessment (Boateng et al., 2018; DeVellis, 2012). In the absence of this information, researchers may incorrectly interpret findings due to measures not adequately reflecting the intended construct.

### Reliability

Tests of reliability were reported in most cases (88%). Cronbach’s alpha was the most commonly used procedure, which is consistent with previous findings (McNeish, 2018). However, the use of Cronbach’s alpha has been criticised for several reasons, including that it is often used as a default without consideration of assumptions, such as a unidimensional structure (Hayes and Coutts, 2020; McNeish, 2018). As such, there is a growing sentiment that coefficient omega is a preferred technique for estimating reliability, (Hayes and Coutts, 2020; McNeish, 2018; Watkins, 2017), but Cronbach’s alpha may still be appropriate in certain circumstances, such as single-factor models with uncorrelated errors (Raykov and Marcoulides, 2019). Pearson’s *r* was the only measure of internal consistency reported for one two-item scale; however, the Spearman-Brown coefficient is recommended for two-item scales, though two-item scales are not recommended as an adequate measure of a construct (Eisinga et al., 2013).

### Dimensionality

Tests of dimensionality were reported in over three-quarters of cases (79%). However, fewer were subjected to EFA (27%). This is important, because without prior information about the latent structure which can be tested with EFA, or a strong theoretical basis that is clearly reflected in the items, researchers may make incorrect assumptions about the dimensionality of a scale, for example, assuming a unidimensional structure when it may be multidimensional (Fabrigar et al., 1999). Several assumptions need to be satisfied for EFA, and it was noted in this review that these were not always satisfied (e.g. some studies did not meet minimum sample size recommendations; Goretzko et al., 2021; Izquierdo et al., 2014). Further, EFA requires decisions and interpretations that affect conclusions regarding dimensionality; therefore, transparent reporting of steps is recommended (Fabrigar et al., 1999; Lloret-Segura et al., 2014), but this was not always present in the articles reviewed (e.g. Johnson et al., 2021). Tests of dimensionality also rely on decisions of the researchers regarding whether items are conceptually related (Fabrigar et al., 1999; Lloret-Segura et al., 2014). Some instances were noted where items were combined into a composite measure, but the literature would suggest these are conceptually distinct (e.g. attitudes towards science vs science knowledge; Allum et al., 2008). Therefore, it is important for researchers to consider whether items reflect the construct of interest when developing or using existing measures.

### Validity

In addition to content validity discussed earlier, the majority of cases reported construct validity (85%), but fewer reported criterion validity (35%). One aspect of criterion validity that is often challenging to assess is concurrent validity, which estimates associations between ‘gold-standard’ measures and scale scores. In the absence of these measures, this aspect of criterion validity cannot be assessed (Boateng et al., 2018). We are not aware of any ‘gold-standard’ measures relevant to attitudes towards science, though this is to be expected given the diverse approaches to measuring this topic. Criterion validity can also be established by estimating whether the instrument predicts other factors that it should be expected to predict (e.g. whether a measure of voting preferences predicts voting behaviours). Given scale development is an iterative process, we acknowledge the possibility that there may be studies where various tests of validity have been conducted, but such studies would have been excluded from this review if a scale was not developed, or refined or evaluated with the reporting of tests of reliability and dimensionality. Therefore, evidence of scale validity may exist in studies not identified in this review.

### Other methodological issues

It should be noted that some papers lacked adequate detail regarding methodology and/or results to make a determination regarding whether the psychometric criteria had been satisfied. For example, Retzbach et al. (2016) reported selectivity and item difficulty statistics in a table, but did not describe their process of item reduction. We recommend that future studies ensure clear and replicable procedures and results are reported.

Overall, these findings highlight the complexities of measuring the varied subconstructs which constitute attitudes towards science. Some strengths of the currently available instruments can be observed from this evidence; however, gaps are also highlighted in terms of adherence to best-practice recommendations, reporting, and continued work to strengthen psychometric properties of instruments.

### Limitations

Several limitations are relevant for this scoping review. In terms of inclusion criteria, articles were limited to those reporting participants aged 18 years and older. It was not anticipated that older adolescents (e.g. aged 15 or 16) are sometimes included in studies examining public attitudes towards science. Although 74 articles were excluded in this review due to age, it should be noted that this criterion was listed as the fourth step of a 10-step exclusion hierarchy, so these articles may not have met subsequent inclusion criteria. To the best of our knowledge, the vast majority of research relating to public attitudes towards science would be targeted at individuals aged 18 years and older, so the risk of relevant articles being excluded is likely low. However, we would encourage future researchers interested in updating this review to consider extending the age range to include participants aged 15 years and older. Further, to limit the size of the review, we restricted articles to those published in English. Therefore, there may be cross-cultural differences that have not been identified in this review. There are other measures beyond the scope of this review that might include questions relevant to attitudes towards science, such as including these questions in large-scale surveys that cover many topics. Our review focused on dedicated efforts to develop instruments that tap into attitudes towards science, so these other measures were not discussed.

In terms of the search strategy, the broad nature of terminology used in the area of attitudes towards science meant that the search strategy was necessarily broad, which presented challenges in terms of the size of the review and logistics. Given this was a large scoping review, we considered limiting the search to particular journals or topics. However, the multidisciplinary nature of this research meant that this option was not desirable, as it may have risked the exclusion of relevant articles, such as those published outside the behavioural and social sciences area. Nevertheless, it would be beneficial to consider additional constraints to the search strategy in future reviews. Further, including search terms for instruments and psychometrics was necessary to narrow the search; however, this may have excluded other valid instruments where these terms were not included in the title or abstract. Though this was a necessary strategy to balance quality with available resources, it would be worth reassessing for future reviews.

## 5. Conclusion

This systematic scoping review identified that there are many and varied conceptualisations of attitudes towards science in the literature, which reflects the complexity of the overarching topic. Promisingly, a number of instruments have been developed following best-practice recommendations for scale development, and have been subjected to more rigorous tests of psychometric properties. This is positive, because this supports the rigour and validity of scientific findings that use these measures. However, other instances highlight gaps in these processes, and the untested assumption of unitary constructs. To continue strengthening rigorous, valid, and evidence-based research methods in fields such as the behavioural and psychological sciences, researchers should aim for transparency when reporting measures (Flake and Fried, 2020). Flake and Fried (2020) propose six questions that researchers can ask themselves when considering reporting of measurement practices: 1. ‘What is the construct?’; 2. ‘Why and how did you select your measure?’; 3. ‘What measures did you use to operationalise the construct?’; 4. ‘How did you quantity your measure?’; 5. ‘Did you modify the scale? And if so, how and why?’; and 6. ‘Did you create a measure on the fly?’ (Flake and Fried, 2020: 459). In addition, researchers should aim to apply recommendations for item development, scale development, and scale evaluation (e.g. AERA et al., 2014; Boateng et al., 2018; DeVellis, 2012). Caution should be applied to ensure appropriate procedures for estimating reliability are selected and assumptions of psychometric tests are met (Fabrigar et al., 1999; Hayes and Coutts, 2020; Lloret-Segura et al., 2014; McNeish, 2018; Watkins, 2017). Further, transparent reporting of necessary steps and statistics should occur. These actions aid in the assessment of the validity of findings, which allows for studies to be replicated and reliable conclusions to be made regarding the body of evidence. By mapping the current instruments that are in use, this scoping review presents an opportunity for future research to focus on further strengthening the psychometric properties of these instruments, including by conducting studies to test their validity. Further, knowledge of the instruments and themes identified in this review will help to facilitate future systematic reviews or meta-analyses examining, for example, the efficacy of interventions designed to modify the constructs measured by these instruments. Ultimately, the outcomes of this scoping review will help to promote robust research and advance our understanding of the important role of attitudes towards science in society.

## Supplemental Material

sj-docx-1-pus-10.1177_09636625261458106 – Supplemental material for Measures of public attitudes towards science: A scoping review Supplemental material, sj-docx-1-pus-10.1177_09636625261458106 for Measures of public attitudes towards science: A scoping review by Jessica E. Hughes, Kimberley Norris, James D. Sauer, Aaron Drummond, Holly Emery, Emilia Hawkey, Victoria J. Heinrich, Paul Schokman and Matthew A. Palmer in Public Understanding of Science

## Appendix 1

**Table 3.** Summary of extracted articles and characteristics of instruments.

Citation	Instrument	Number of items	Response scaling	Response options	Population	Study setting
Besley et al. (2006)	Perceived Justice of Local Science Authorities	12 items overall; 3 items for each of the 4 subscales	5-point Likert scale	Strongly disagree, somewhat disagree, don’t know/unsure, somewhat agree, strongly agree	*N* = 1305; Adults	General public (US)
Boschetti et al. (2012)	Attitude Towards Science	5	5-point Likert scale	Disagree strongly, disagree, neutral, agree, agree strongly	*N* = 250; Adults aged 18 years and older (Additional stakeholder sample *N* = 17).	General public (Australia)
Calnan et al. (2005)	Beliefs About Science	8	5-point Likert scale	Strongly agree, agree, neither agree or disagree, disagree, strongly disagree	*N* *=* 1187; Adults aged 18 and older	General public; England and Wales
Crescenzi-Lanna et al. (2024)	Scepticism About Science	9	4-point Likert scale	Strongly agree; agree; disagree; strongly disagree	*N* = 1128; Adults aged 18-25 years	General public (Italy)
Crescenzi-Lanna et al. (2024)	Unrealistic Expectations About Science	8	4-point Likert scale	Strongly agree; agree; disagree; strongly disagree	*N* = 1128; Adults aged 18-25 years	General public (Italy)
Dagnall et al. (2019)	Belief in Science Scale (BISS)	10	6-point Likert scale	1 – Strongly disagree to 6 – Strongly agree	Study 1: *N* *=* 686; Adults aged 18-69 years. Study 2: *N* = 534; Adults aged 18-71 years.	University students, university staff, general public (UK)
Drummond et al. (2016)	Endorsement of Scientific Inquiry	8	7-point Likert scale	1 – Strongly disagree to 7 – Strongly agree	*N* *=* 215; Adults	General public (US)
Einsiedel (1994)	Attitudes Towards Science and Technology	10	Not reported	Not reported	*N* = 2000 (response rate 76%); Adults	General public (Canada)
Farias et al. (2013)	Belief in Science Scale (BISS)	10	6-point Likert scale	1 – Strongly disagree to 6 – Strongly agree	Study 1: *N* = 144; Aged 18 years and older	Not reported
Hartman et al. (2017)	Credibility of Science Scale	6	7-point Likert scale	1 – Disagree very strongly to 7 – Agree very strongly (all reverse coded)	Study 1: *N* *=* 525; Adults aged 18-89. Study 2: *N* = 1436; Adults aged 18-84. Study 3: *N* = 600; Adults aged 18-84.	General public (country not specified)
Howell et al. (2020)	Belief in the Authority of Science	2	7-point Likert scale	1 – Strongly disagree to 7 – Strongly agree	*N* = 1517; Adults	General public (US)
Jach (2019)	Views of Science Questionnaire	16	5-point Likert scale	1 – Definitely disagree, 2 – Rather disagree, 3 – Difficult to say, 4 – Rather agree, 5 – Definitely agree	Studies 1–3: *N* = 508 (EFA = 254; CFA = 254–380); Adults aged 18 years and older. Study 4: *N* = 100	Studies 1–3: General public (country not specified). Study 4: University students (Poland)
Jach (2021)	Views of Science Questionnaire	16	5-point Likert scale	1 – Definitely disagree to 5 – Definitely agree	*N* = 1119; Adults aged 18 to 87.	General public (Poland)
Johnson et al. (2021)	Science Mindset	5	7-point Likert scale	1 – Strongly disagree to 7 – Strongly agree	*N* = 858; Adults	General public (US)
Kahan et al. (2017)	Science Curiosity Scale	12	Various	Various	Study 1: *N* = 2500; Study 2: *N* = 3000. Adults	General public (country not specified)
Kawamoto et al. (2013)	Science-Appreciating Scale	8	4-point Likert scale	Agree, slightly agree, slightly disagree, disagree	*N* = 1286; Adults aged 18-69.	General public (Japan)
Lewandowsky and Oberauer (2021)	Norms of Science	14	7-point Likert scale	1 – Strongly disagree to 7 – Strongly agree	Study 2: *N* = 1038; Adults aged 18-82.	General public (US)
Lombrozo et al. (2008)	Nature of Science	60	5-point Likert scale	Strongly disagree, disagree, neither agree nor disagree, agree, strongly agree	*N* = 96; Adults	University students (US)
Lukić and Žeželj (2024)	Scientific Beliefs Questionnaire (Uncritical Trust in Science)	12	Thurstone scale	5 graded response options	Study 1: *N* = 200; Adults aged 18 years and older. Study 2: *N* = 383; Adults aged 18 years and older.	General public (Serbia)
Morgan et al. (2018)	Negative Perceptions of Science Scale	20	Not reported	Not reported	Study 1: *N* (EFA) = 251; *N* (CFA) = 251; Adults aged 18 years and older. Study 2	General public (US)
Motta et al. (2021)	Science Curiosity Scale – Random Subset (RS)	Random selection of 67% of original 12 Science Curiosity Scale items.	Various	Various	Study 1: *N* = 2500; Adults. Study 2: *N* = 3000; Adults.	General public (US)
Motta et al. (2021)	Science Curiosity Scale – Reduced-Form (RF)	4	Various	Various	Study 1: *N* = 2500; Adults. Study 2: *N* = 3000; Adults.	General public (US)
Neumann et al. (2011)	Nature of Science and Scientific Inquiry	23	5-point Likert scale	Strongly agree, agree, not sure, disagree, strongly disagree	*N* = 214; Adults aged 19-31.	University students (US)
Park et al. (2024)	Belief in Science Scale (BISS)	7 (of original 10)	6-point Likert scale	1 – Strongly disagree to 6 – Strongly agree	*N* = 300; Adults aged 18 years and older.	General public (US)
Price and Lee (2013)	Nature of Scientific Knowledge Scale (NSKS)	24	5-point Likert scale	Strongly disagree, disagree, neutral, agree, strongly agree	*N* = 333; Adults aged 18-64	Citizen science volunteers (country not reported)
Price and Lee (2013)	Scientific Attitude Instrument	9	5-point Likert scale	Strongly disagree, disagree, neutral, agree, strongly agree	*N* = 3180 (pre), 333 (post); Adults aged 18-64	Citizen science volunteers (country not reported)
Retzbach et al. (2016)	Perceived Uncertainty of Scientific Evidence	10	6-point Likert scale	Strongly disagree to strongly agree	Study 1: *N* *=* 502 (Germany), *N* = 492 (US); Adults aged 18 years and older. Study 2: *N* = 587 (US); Adults	General public (Germany and US)
Schoor and Schütz (2021)	Utility of Science	4	5-point Likert scale	1 – Not agree at all, 2 – Rather not agree, 3 – Draw, 4 – Rather agree, 5 – Fully agree	*N* = 261; Adults aged 18-68	University students (Germany)
Shin et al. (2024)	Reasoning through Evidence Versus Advice (EvA) Scale	16	7-point Likert scale	Strongly disagree to strongly agree	Study 1: *N* *=* 549; Adults aged 18 years and older. Study 2: *N* = 189; Adults aged 18 years and older. Study 3: *N* = 316; Adults aged 18 years and older. Study 4: *N* = 529; Adults aged 18 years and older.	General public (US)
Tavani et al. (2021)	Credibility of Science Scale	6	7-point Likert scale	Disagree very strongly, disagree strongly, disagree somewhat, neither agree nor disagree, agree somewhat, agree strongly, agree very strongly	Study 1a: *N* = 393; Adults aged 18-58 years. Study 1b: *N* = 302; Adults aged 18-74 years. Study 1c: *N* = 320; Adults aged 18-72 years. Study 2: 319; Adults aged 18-72 years.	General public (France)
Vaccarezza (2007)	Valuation of Science and Technology	18	Likert scale	Not reported	*N* = 300; Adults aged 18 years and older.	General public (Argentina)
Valdesolo et al. (2016)	Belief in Scientific Order	2	6-point Likert scale	Tremendously doubtful, doubtful, slightly doubtful, slightly likely, likely, extremely likely	*N* = 364; Adults	General public/university (country not specified)
Weisberg et al. (2021)	Nature of Science Index	20	5-point Likert scale	Strongly disagree, disagree, unsure, agree, strongly agree	*N* = 1500; Adults aged 18-92 years	General public (US)
Winter et al. (2022)	Credibility of Science Scale	6	7-point Likert scale	1 – Strongly disagree to 7 – Strongly agree	*N* = 888; Average age 49.7	General public (New Zealand)

*N* = sample size; EFA = exploratory factor analysis; CFA = confirmatory factor analysis.

## Appendix 2

**Table 4.** Psychometric properties of the instruments identified after extraction.

Citation	Instrument	Construct	Psychometric properties							
			Theoretical background	Item development	Item reduction	Dimensionality	Reliability	Content validity	Criterion validity	Construct validity
Besley et al. (2006)	Perceived Justice of Local Science Authorities	Perceived distributive, procedural, interpersonal, and informational justice relating to scientific research and scientists within local communities.	Yes	Unclear	Not reported	EFA	α range from .63 to .91 across subscales	Not reported	Yes	Yes
Boschetti et al. (2012)	Attitude Towards Science	Belief in science in general and as a way of solving natural/environmental and social problems.	Unclear	Unclear	Not reported	Not reported	α = .75	Not reported	Not reported	Yes
Calnan et al. (2005)	Beliefs About Science	Attitudes towards scientific knowledge, risks associated with science, scientists, and interest in science.	Unclear	Yes	Not reported	Not reported	α = .78	Yes	Not reported	Yes
Crescenzi-Lanna et al. (2024)	Scepticism About Science	Attitudes towards the importance, influence, and credibility of science and scientists.	Yes	Yes	Not reported	PCA; CFA	α = .74	Not reported	Yes	Yes
Crescenzi-Lanna et al. (2024)	Unrealistic Expectations About Science	Attitudes towards the acceptability of debate and uncertainty within science and among scientists and the communication of uncertainty with the public.	Yes	Yes	Not reported	PCA; CFA	α = .78	Not reported	Not reported	Yes
Dagnall et al. (2019)	Belief in Science Scale (BISS)	Value for the institution of science and superiority of scientific knowledge.	Yes	Yes	Unclear	EFA; CFA	Ω = .91 (EFA); Ω = .93 (CFA)	Not reported	Not reported	Yes
Drummond et al. (2016)	Endorsement of Scientific Inquiry	Endorsement of scientific research and the processes of scientific inquiry as a way of accumulating knowledge, making decisions, and solving problems.	Yes	Yes	Not reported	Not reported	α = .79	Not reported	Not reported	Yes
Einsiedel (1994)	Attitudes Towards Science and Technology	Attitudes towards science and scientists in relation to society.	Yes	Unclear	Not reported	EFA	Not reported	Not reported	Not reported	Yes
Farias et al. (2013)	Belief in Science Scale (BISS)	Value for the institution of science and superiority of scientific knowledge.	Yes	Yes	Not reported	EFA; PCA	α = .86	Not reported	Not reported	Yes
Hartman et al. (2017)	Credibility of Science Scale	Evaluation of scientific methods, information, and the institution of science in terms of perceived accuracy, usefulness, and motives.	Yes	Yes	Yes	EFA; CFA; IRT	α = .95	Not reported	Yes	Yes
Howell et al. (2020)	Belief in the Authority of Science	Belief in science as a way of knowing and understanding the world.	Yes	Yes	Yes	Not reported	Pearson’s *r* = .75	Not reported	Not reported	Yes
Jach (2019)	Views of Science Questionnaire	Scientistic worldview including trust in science, the authority of science and scientists, science as a basis for unity and hope, and the application of science.	Yes	Yes	Yes	EFA; CFA	α ranged from .62 to .87 across studies/subscales; test-retest	Not reported	Not reported	Yes
Jach (2021)	Views of Science Questionnaire	Scientistic worldview including trust in science, the authority of science and scientists, science as a basis for unity and hope, and the application of science.	Yes	Yes	Yes	CFA; Other	α = .93 overall; subscales ranged from .78 to .87	Not reported	Not reported	Yes
Johnson et al. (2021)	Science Mindset	Belief in the use of logic and scientific processes to understand, explain, and address important challenges.	Yes	Yes	Not reported	PCA	α = .91	Not reported	Yes	Yes
Kahan et al. (2017)	Science Curiosity Scale	Interest in science-based media and activities for personal enjoyment.	Yes	Yes	Not reported	IRT	IRT	Not reported	Yes	Yes
Kawamoto et al. (2013)	Science-Appreciating Scale	Attitudes towards science and scientists in relation to society.	Yes	Yes	Yes	EFA; Other	Not reported	Unclear	Not reported	Yes
Lewandowsky and Oberauer (2021)	Norms of Science	Views about how scientific research should be conducted, scientific findings should be used, and scientists should conduct themselves.	Yes	Yes	Not reported	SEM	Ω = .66	Not reported	Not reported	Yes
Lombrozo et al. (2008)	Nature of Science	Attitudes towards the nature of scientific inquiry and the role of science in society.	Yes	Yes	Not reported	PCA	Not reported	Not reported	Yes	Yes
Lukić and Žeželj (2024)	Scientific Beliefs Questionnaire (Uncritical Trust in Science)	Belief in the nature of science as the only pathway to knowledge and truth, which is fundamentally valuable and beneficial to all.	Yes	Yes	Not reported	PCA; CFA	α ranged from .75 to .84 across studies	Yes	Not reported	Yes
Morgan et al. (2018)	Negative Perceptions of Science Scale	Belief that science and scientists are corrupt, onerous, heretical, and limited.	Yes	Yes	Yes	EFA; CFA	α ranged from .71 to .96 across subscales/studies	Not reported	Not reported	Yes
Motta et al. (2021)	Science Curiosity Scale–Random Subset (RS)	Interest in science-based media and activities for personal enjoyment.	Yes	Yes	Yes	IRT	IRT (> .70)	Yes	Yes	Yes
Motta et al. (2021)	Science Curiosity Scale–Reduced-Form (RF)	Interest in science-based media and activities for personal enjoyment.	Yes	Yes	Yes	IRT	IRT (ranged from .60 to .80)	Yes	Yes	Yes
Neumann et al. (2011)	Nature of Science and Scientific Inquiry	Attitudes towards the nature of scientific inquiry and the role of science in society.	Yes	Yes	Yes	IRT	α ranged from .35 to .89 across subscales/versions	Not reported	Not reported	Not reported
Park et al. (2024)	Belief in Science Scale (BISS)	Value for the institution of science and superiority of scientific knowledge.	Yes	Yes	Yes	CFA; Other	α = .95 (items 3 to 9 of the BiSS)	Not reported	Not reported	Yes
Price and Lee (2013)	Nature of Scientific Knowledge Scale (NSKS)	Attitudes towards the nature of scientific inquiry.	Yes	Yes	Yes	Not reported	α = .94; IRT	Not reported	Not reported	Not reported
Price and Lee (2013)	Scientific Attitude Instrument	Interest in science-based media and activities for personal enjoyment, and personal knowledge and application of science information.	Unclear	Yes	Yes	Not reported	α = .95; IRT	Not reported	Yes	Not reported
Retzbach et al. (2016)	Perceived Uncertainty of Scientific Evidence	Perceptions regarding the extent to which scientific knowledge is uncertain and subject to change.	Yes	Yes	Unclear	EFA; CFA	α = .75 (Subjective subscale); α = .87 (Objective subscale)	Yes (German version)	Not reported	Yes
Schoor and Schütz (2021)	Utility of Science	Attitudes towards the usefulness of scientific information in decision-making.	Yes	Yes	Not reported	CFA	Ω = .79 (Utility of Science); Ω = .76 (Utility of Personal Experiences)	Not reported	Yes	Yes
Shin et al. (2024)	Reasoning through Evidence Versus Advice (EvA) Scale	Inclination to source and trust evidence, and make decisions based on expert advice.	Yes	Yes	Yes	EFA; CFA	α ranged from .68 to .85 across subscales; Ω ranged from .68 to .85 across subscales	Yes	Yes	Yes
Tavani et al. (2021)	Credibility of Science Scale	Evaluation of scientific methods, information, and the institution of science in terms of perceived accuracy, usefulness, and motives.	Yes	Yes	Not reported	PCA; CFA	α ranged from .80 to .90 across studies; Other	Not reported	Yes	Yes
Vaccarezza (2007)	Valuation of Science and Technology	Attitudes towards the risks and benefits of science, technology, and scientists in relation to society.	Not reported	Unclear	Unclear	EFA	Not reported	Not reported	Not reported	Not reported
Valdesolo et al. (2016)	Belief in Scientific Order	Belief in science as a way of explaining events and providing order.	Not reported	Yes	Yes	Not reported	α = .69	Not reported	Not reported	Not reported
Weisberg et al. (2021)	Nature of Science Index	Attitudes towards the nature of scientific inquiry.	Yes	Unclear	Not reported	EFA	α = .84	Not reported	Not reported	Yes
Winter et al. (2022)	Credibility of Science Scale	Evaluation of scientific methods, information, and the institution of science in terms of perceived accuracy, usefulness, and motives.	Not reported	Yes	Not reported	CFA	α = .90	Not reported	Not reported	Yes

EFA = exploratory factor analysis; CFA = confirmatory factor analysis; PCA = principal components analysis; IRT = item response theory; SEM = structural equation modelling; α = Cronbach’s alpha; Ω = McDonald’s omega. Not reported = information relating to category not reported in article; Unclear = partial reporting of information in article, but with insufficient detail to satisfy requirements for the category; Yes = information relating to category has been reported in article.

## Notes

1. It should be noted that the original protocol specified that we were conducting a systematic review; however, shortly after commencing the review, we realised that our aims more closely aligned with a scoping review, so the type of review was changed accordingly.

2. Although we use the word ‘towards’ throughout this manuscript, we specifically excluded this from the search terms due to grammatical differences between UK and US.
