# Supplementary material for: Measures of public attitudes towards science: A scoping review
Source: Public Underst Sci. 2026 Jul 12;35(7):891–916. doi: 10.1177/09636625261458106 (PMC13598314; doi:10.1177/09636625261458106)
Supplement: Supplementary file 1 [file sj-docx-1-pus-10.1177_09636625261458106.docx]

Supplemental Material for

Measures of Public Attitudes Towards Science:

A Scoping Review

*Jessica E. Hughes^1^: jessica.hughes@utas.edu.au

Kimberley Norris: kimberley.norris@utas.edu.au

James D. Sauer^1^: jim.sauer@utas.edu.au

Aaron Drummond^1^: aaron.drummond@utas.edu.au

Holly Emery^1^: holly.emery@utas.edu.au

Emilia Hawkey^1^: emilia.hawkey@utas.edu.au

Victoria J. Heinrich^1^: vicki.heinrich@utas.edu.au

Paul Schokman^1^: paul.schokman@utas.edu.au

Matthew A. Palmer^1^: matthew.palmer@utas.edu.au

^1^ School of Psychological Sciences, University of Tasmania, Tasmania, Australia

Supplemental Table 1 shows the search terms entered across the three databases used in this scoping review. The structure of the search was consistent, such that terms were located in titles and abstracts, but search terms were adapted to suit the individual databases.

**Supplemental Table 1**

*Search terms entered into databases used to access scoping review literature*

| Database | Search terms |
| --- | --- |
| Scopus | ( ( ( ( TITLE ( scien* W/2 ( attitud* OR valu* OR belief* OR endors* OR support* OR faith OR trust OR concern* OR respect* OR regard OR credib* OR interest* OR curio* OR accept* ) ) ) AND ( TITLE ( scale* OR instrument* OR measure* OR survey* OR questionnaire* OR tool* OR test* ) ) AND ( TITLE ( valid* OR develop* OR psychometric OR analys* OR construct* OR reliab* OR sensitiv* ) ) ) OR ( ( ABS ( scien* W/2 ( attitud* OR valu* OR belief* OR endors* OR support* OR faith OR trust OR concern* OR respect* OR regard OR credib* OR interest* OR curio* OR accept* ) ) ) AND ( ABS ( scale* OR instrument* OR measure* OR survey* OR questionnaire* OR tool* OR test* ) ) AND ( ABS ( valid* OR develop* OR psychometric OR analys* OR construct* OR reliab* OR sensitiv* ) ) ) ) AND NOT ( TITLE-ABS ( secondary OR elementary OR "high school" OR "primary school" OR "middle school" OR teacher* OR student* OR child* OR pupil* OR parent* ) ) ) AND ( LIMIT-TO ( DOCTYPE,"ar" ) ) AND ( LIMIT-TO ( LANGUAGE,"English" ) ) |
| PsychINFO | 1. (scien* adj2 (attitud* or valu* or belief* or endors* or support* or faith or trust or concern* or respect* or regard or credib* or interest* or curio* or accept*)).ab,ti.  2. (valid* or develop* or psychometric* or analys* or construct* or reliab* or sensitiv*).ab,ti.  3. exp Psychometrics/  4. 2 or 3  5. (scale* or instrument* or measure* or survey* or questionnaire* or tool* or test*).ab,ti.  6. 1 and 4 and 5  7. (secondary or elementary or “high school” or “primary school” or “middle school” or teacher* or student* of child* or pupil*).ab,ti.  8. 6 not 7 |

**Supplemental Table 1 (cont.)**

| Database | Search terms |
| --- | --- |
| Web of Science | 1. TI=((scien* NEAR/2 (attitud* or valu* or belief* or endors* or support* or faith or trust or concern* or respect* or regard or credib* or interest* or curio* or accept*)))  2. TI=(scale* OR instrument* OR measure* OR survey* OR questionnaire* OR tool* OR test*)  3. TI=((valid* OR develop* OR psychometric))  4. #1 AND #2 AND #3  5. AB=((scien* NEAR/2 (attitud* OR valu* OR belief* OR endors* OR support* OR faith OR trust OR concern* OR respect* OR regard OR credib* OR interest* OR curio* OR accept*)))  6. AB=((scale* OR instrument* OR measure* OR survey* OR questionnaire* OR tool* OR test*))  7. AB=((valid* OR develop* OR psychometric* OR analys* OR construct* OR reliab* OR sensitiv* ))  8. #5 AND #6 AND #7  9. #4 OR #8  10. TI=(secondary OR elementary OR "high school" OR "primary school" OR "middle school" OR teacher* OR student* OR child* OR pupil*)  11. AB=(secondary OR elementary OR "high school" OR "primary school" OR "middle school" OR teacher* OR student* OR child* OR pupil*)  12. #10 OR #11  13. #9 NOT #12 |
